# Comparative adaptations in oxidative and glycolytic muscle fibers in a low voluntary wheel running rat model performing three levels of physical activity

**DOI:** 10.14814/phy2.12619

**Published:** 2015-11-24

**Authors:** Hayden W Hyatt, Ryan G Toedebusch, Greg Ruegsegger, C Brooks Mobley, Carlton D Fox, Graham R McGinnis, John C Quindry, Frank W Booth, Michael D Roberts, Andreas N Kavazis

**Affiliations:** 1 School of Kinesiology, Auburn UniversityAuburn, Alabama; 2 Department of Biomedical Sciences, University of MissouriColumbia, Missouri

**Keywords:** Antioxidants, exercise, sedentary

## Abstract

A unique polygenic model of rat physical activity has been recently developed where rats were selected for the trait of low voluntary wheel running. We utilized this model to identify differences in soleus and plantaris muscles of sedentary low voluntary wheel running rats and physically active low voluntary wheel running rats exposed to moderate amounts of treadmill training. Three groups of 28-day-old male Wistar rats were used: (1) rats without a running wheel (SEDENTARY, *n* = 7), (2) rats housed with a running wheel (WHEEL, *n* = 7), and (3) rats housed with a running wheel and exercised on the treadmill (5 days/week for 20 min/day at 15.0 m/min) (WHEEL + TREADMILL, *n* = 7). Animals were euthanized 5 weeks after the start of the experiment and the soleus and plantaris muscles were excised and used for analyses. Increases in skeletal muscle gene expression of peroxisome proliferator-activated receptor gamma coactivator 1 alpha and fibronectin type III domain-containing protein 5 in WHEEL + TREADMILL group were observed. Also, WHEEL + TREADMILL had higher protein levels of superoxide dismutase 2 and decreased levels of oxidative damage. Our data demonstrate that the addition of treadmill training induces beneficial muscular adaptations compared to animals with wheel access alone. Furthermore, our data expand our understanding of differential muscular adaptations in response to exercise in mitochondrial, antioxidant, and metabolic markers.

## Introduction

Low daily physical activity is associated with obesity and numerous metabolic maladies. Obese, physically inactive people are at increased risk for more than 35 clinically relevant poor health conditions and premature death (Bauman et al. 2012). The prevention of body weight gain by increasing physical activity is a key strategy to prevent the global epidemic of obesity (Caballero 2007). However, 58% of children (6–11 years of age) and about 92% of adolescents (12–19 years) do not meet recommended doses of daily physical activity (Troiano et al. 2008). Chronic diseases brought on by physical inactivity are effectively prevented by exercise and benefits are manifested in a positive dose–response relationship (Warburton et al. 2006).

The functional plasticity of skeletal muscle evokes a phenotypic continuum that undergoes constant physiological adaptations in response to its environment. As such, it is important to note that low daily physical activity levels are associated with a more glycolytic skeletal muscle phenotype (Short et al. 2005) and high levels of daily physical activity promote a more oxidative skeletal muscle fiber type (Holloszy and Booth 1976) as well as an increase in intramuscular mitochondrial content (Holloszy 1975) and antioxidants (Powers et al. 1999). Furthermore, physical activity increases the intramuscular expression of putative myokines (e.g., fibronectin domain-containing protein 5 [FNDC5]) which act to further promote skeletal muscle plasticity toward a healthier phenotype (Pedersen 2013; Seo et al. 2014).

Booth and collaborators recently used selective breeding to develop rats that voluntarily run low nightly distances (termed low voluntary runners) (Roberts et al. 2012, 2013, 2014; Brown et al. 2015). While it is challenging to relate rodent physiology to human physiology, low voluntary wheel running rats may approximate inactive human lifestyles. Given the uniqueness of selectively bred model of voluntary physical activity, we examined whether increased physical activity in low voluntary wheel running rats elicits adaptive changes in skeletal muscle. Specifically, we hypothesized that extended physical activity exposure in low voluntary wheel running rats through supplemental treadmill running would alter select metabolic skeletal muscle markers. Differences in soleus and plantaris muscles fiber type, select metabolic and mitochondrial markers, and oxidative stress were examined in these rats following a 5-week experimental protocol.

## Methods

### Animals and selective breeding

The Institutional Animal Care and Use Committee at the University of Missouri-Columbia approved all animal experiments. A selective breeding model was performed similar to that of Swallow et al. (1998) and Koch and Britton (2001) and has been described in detail previously (Roberts et al. 2012, 2013). For the current experiments, male rats (28 days old) from generation 11 (G11) of the LVR line were assigned to one of the three groups: (1) SEDENTARY (*n* = 7), rats without access to a running wheel in their home cage; (2) WHEEL (*n* = 7), rats with access to a running wheel in their home cage; and (3) WHEEL + TREADMILL (*n* = 7), rats with access to a running wheel in their home cage in addition to exercising on a treadmill for 20 min a day, 5 days a week, at a speed of 15 m/min for 5 weeks (about 40% VO_2max_).

All animals were maintained in temperature-controlled quarters (21°C) with a 12:12-h light:dark cycle, and all animals were provided standard chow and water ad libitum. Following the experimental period, animals were euthanized using CO_2_. Soleus and plantaris skeletal muscles were immediately harvested and flash frozen in liquid nitrogen and stored at −80°C until analysis, and the contralateral leg skeletal muscles were mounted on cork, frozen in liquid nitrogen-cooled isopentane, and stored at −80°C until histochemical analysis.

### Muscle fiber typing

Serial sections from soleus and plantaris samples were cut at 10 *μ*m using a cryotome (HM 525 Cryostat; Thermo Fisher Scientific, Waltham, MA). Sections were dried at room temperature for 30 min and incubated in a phosphate-buffered saline (PBS) solution containing 0.5% Triton X-100. Sections were rinsed in PBS and subsequently exposed to primary antibodies specific to dystrophin protein (rabbit host, # RB9024R7; Lab Vision Corporation, Fremont, CA), myosin heavy chain Type I (mouse host, immunoglobulin M [IgM] isotype, # A4.840; Developmental Studies Hybridoma Bank, Iowa City, IA), and myosin heavy chain Type IIa (mouse host, immunoglobulin G [IgG] isotype, # SC71; Developmental Studies Hybridoma Bank) in a dark humid chamber at room temperature for 1 h. Sections were subsequently rinsed three times in PBS and exposed to rhodamine red anti-rabbit secondary antibody (R6394; Molecular Probes, Eugene, OR), Alexa Fluor 350 goat anti-mouse IgM isotype-specific secondary antibody (# A31552; Molecular Probes), and Alexa Fluro 488 goat anti-mouse IgG isotype-specific secondary antibody (A11011; Molecular Probes) diluted in PBS containing 0.5% Pierce Super Blocker (Thermo Fisher Scientific) in a dark humid chamber at room temperature for 1 h. Sections were washed in PBS and viewed via a fluorescence microscope (Nikon Instruments, Melville, NY). Fiber typing utilizing this method allows for the individual visualization of the myofiber membrane protein dystrophin using the rhodamine filter set (red), Type I myosin using the DAPI (4′,6-diamidino-2-phenylindole) filter set (blue), Type IIa myosin using the FITC (fluorescein isothiocyanate) filter set (green), and Type IIb/IIx fibers (nonstained/black) myofibers. Images were obtained at a 10× magnification, were merged using NIS-Elements software (Nikon Instruments), and myofibers were analyzed for percent of each MHC by a blinded investigator.

### RT-PCR for skeletal muscle mRNA expression

RNA was isolated from soleus and plantaris using the Ribozol method (Amresco, Solon, OH) according to the manufacturer’s instructions. Concentration and purity of the extracted RNA were measured spectrophotometrically at 260 and 280 nm using the NanoDrop Lite Spectrophotometer (Thermo Fisher Scientific). Following isolation, 1 *μ*g of RNA was reverse transcribed into cDNA using a cDNA synthesis kit (Quanta, Gaithersburg, MD) as per manufacturer’s recommendations. Real-time PCR was performed using SYBR green chemistry (Quanta) with gene-specific primers: glucose transporter type 4 (GLUT4), forward primer 5′-GCTTCTGTTGCCCTTCTGTC-3′ and reverse primer 5′-TGGACGCTCTCTTTCCAACT-3′; brain-derived neurotrophic factor (BDNF), forward primer 5′-GGACTTGTACACTTCCCGGG-3′ and reverse primer 5′-ATGTTTGCGGCATCCAGGTA-3′; FNDC5, forward primer 5′-CCTGGACACAGGGTGAAAGT-3′ and reverse primer 5′-CAAAGAGGAAGCCAGTCCAG-3′; carnitine palmitoyltransferase 1B (CPT1B), forward primer 5′-GCAAACTGGACCGAGAAGAG-3′ and reverse primer 5′-CCTTGAAGAAGCGACCTTTG-3′; peroxisome proliferator-activated receptor gamma coactivator 1-alpha (PGC-1α), forward primer 5′-ATGTGTCGCCTTCTTGCTCT-3′ and reverse primer 5′-ATCTACTGCCTGGGGACCTT-3′; uncoupling protein 3 (UCP3), forward primer 5′-GAGTCAGGGGACTGTGGAAA-3′ and reverse primer 5′-GCGTTCATGTATCGGGTCTT-3′; mitochondrial transcription factor A (TFAM), forward primer 5′-ATCAAGACTGTGCGTGCATC-3′ and reverse primer 5′-AAAGCCCGGAAGGTTCTTAG-3′; and beta-actin, forward primer 5′-GTGGATCAGCAAGCAGGAGT-3′ and reverse primer 5′-ACGCAGCTCAGTAACAGTCC-3′.

Primer efficiency curves for all genes were generated, and efficiencies ranged between 90% and 110%. Relative quantification of gene expression was performed using the comparative CT method. This method uses a single calibrator sample for comparison of every unknown sample’s gene expression. ∆∆CT[∆CT(calibrator) − ∆CT(sample)] was then calculated for each sample and relative quantification was calculated as 2∆∆CT. Beta-actin was used as the reference gene based on previous work showing unchanged expression with our experimental manipulations (Roberts et al. 2013).

### Western blot analysis

Soleus and plantaris muscles were homogenized 1:10 (wt/vol) in 5 mmol/L Tris (pH 7.5) and 5 mmol/L EDTA (pH 8.0) with a protease inhibitor cocktail (Sigma, St Louis, MO) and centrifuged at 1500 *g* for 10 min at 4°C. The resulting supernatant (cytosolic) fraction was collected and protein content was assessed by the method of Bradford (Sigma). Equal amount of proteins were separated by polyacrylamide gel electrophoresis via 12% polyacrylamide gels containing 0.1% sodium dodecyl sulfate for ∼2 h at 120 V (C.B.S. Scientific Company, San Diego, CA). After electrophoresis, the proteins were transferred to polyvinylidene difluoride membranes (Amresco) via the C.B.S. Scientific Company system for 2 h at 200 mA. Nonspecific sites were blocked for 1 h at room temperature in PBS solution containing 0.05% Tween and 5% nonfat milk. Membranes were then incubated for 1 h with primary antibodies directed against the proteins of interest. The primary antibodies used were superoxide dismutase 2 (SOD2; # GTX116093; GeneTex, Irvine, CA), catalase (# GTX110704; GeneTex), and 4-hydroxynonenal-conjugated proteins (4-HNE, # ab46545; Abcam, Cambridge, MA). Alpha tubulin (#12G10; Developmental Studies Hybridoma Bank) was used as the normalizing control. Following incubation with primary antibodies, membranes were washed extensively with PBS–Tween and then incubated with secondary antibodies. Membranes were then developed using an enhanced chemiluminescent reagent (Amersham, Pittsburgh, PA), and band densitometry was performed through the use of a UVP Imager and associated densitometry software (UVP, LLC, Upland, CA).

### Statistical analysis

Comparisons between groups for each dependent variable were made by using a two-tailed one-way analysis of variance (ANOVA) and, when appropriate, a Tukey honestly significant difference test was performed post hoc. Significance was established at *P *<* *0.05. Data are presented as mean ± SEM.

## Results

### Voluntary wheel running effects on body weight, running distance, and myosin heavy chain fiber type

**Figure 1:**
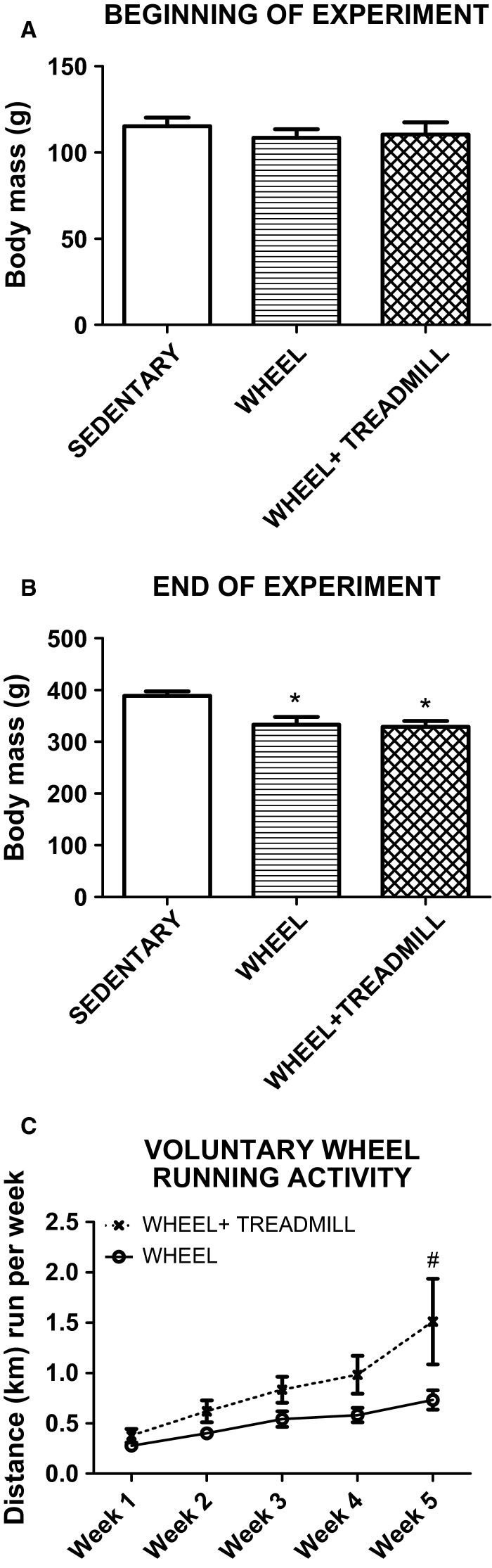
Body mass and wheel running activity of the experimental animals. *Different from SEDENTARY. ^#^Different from WHEEL. *n* = 7/group.

Body weight measurements were determined before and after the 5 weeks of running or sedentary lifestyle. At the beginning of the experiment, all animals expressed no significant differences in body mass (Fig.1A). However, at the end of the 5 weeks of the experimental protocol, body mass in groups with access to a running wheel were significantly (*P* < 0.05) lower compared to SEDENTARY (Fig.1B). To examine any potential additional stimulus of treadmill training in WHEEL + TREADMILL, nightly running wheel activity was measured for the duration of the 5 weeks (Fig.1C). Interestingly, the treadmill intervention in WHEEL + TREADMILL animals resulted in a significant increase (*P* < 0.05) in voluntary wheel running activity in week 5 of training compared to WHEEL.

**Figure 2:**
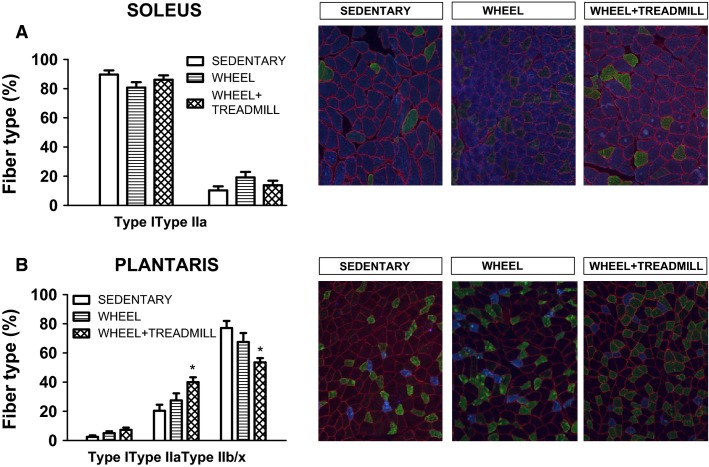
Myosin heavy chain isoform expression in soleus and plantaris. Representative images are shown to the right-hand side of the graphs. Fiber membranes are highlighted by dystrophin stain (red). Fiber types can be identified as MHC I (blue), MHC IIa (green), and all remaining fibers were classified as MCH IIb/x (black). *Different from SEDENTARY. *n* = 6–7/group.

Whether increased physical activity can alter myosin heavy chain fiber type remains controversial (Booth and Thomason 1991). In the current experiment, treadmill training resulted in fiber-type differences in plantaris muscle but not soleus muscle. Specifically, in plantaris, WHEEL + TREADMILL animals had a lower percentage of Type IIb/x fibers and a higher percentage of Type IIa fibers compared to SEDENTARY (*P* < 0.05) (Fig.2B). No significant differences in fiber type existed between solei for all groups (Fig.2A).

### Metabolic adaptations

**Figure 3:**
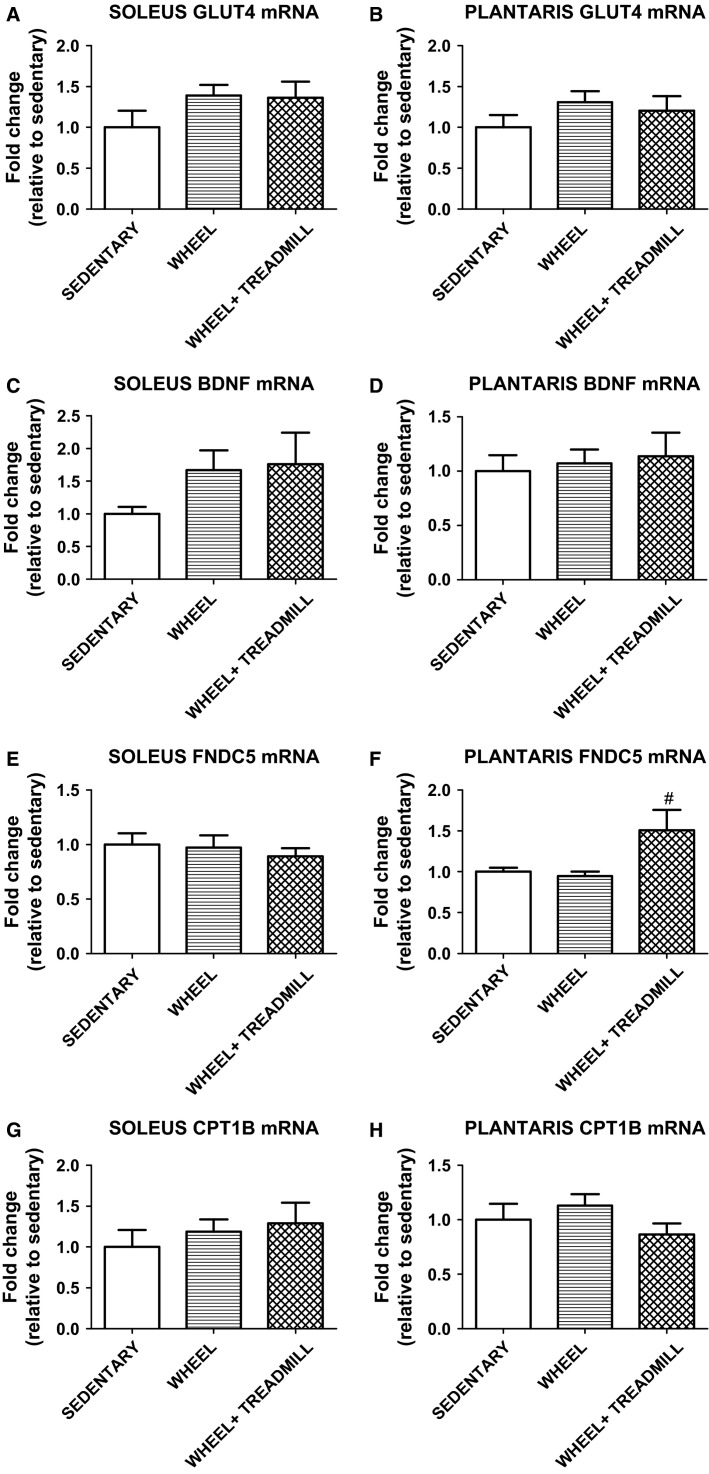
Expression of mRNA of select markers of muscle metabolism in soleus and plantaris. GLUT4 = glucose transporter type 4; BDNF = brain-derived neurotrophic factor; FNDC5 = fibronectin type III domain-containing protein 5; CPT1B = carnitine palmitoyltransferase 1B. ^#^Different from WHEEL. *n* = 6–7/group.

Myokines are hormone-like substances and can play an integral role in metabolic function (Pedersen 2009, 2013). In order to elucidate the effect that varying levels of physical activity had on myokines, the relative expression of key mRNAs were measured in the soleus and plantaris muscles. BDNF can increase lipid oxidation (Matthews et al. 2009). In the current study, there were no significant differences in BDNF mRNA expression in any group (Fig.3C and D). Alterations in adipose tissue composition can also be affected by myokines (Quinn et al. 2009). For example, FNDC5 is an upstream mediator for stimulating the browning of adipose tissue which results in an increase in total body energy expenditure (Bostrom et al. 2012). Our results indicate a significant increase (*P* < 0.05) in FNDC5 in plantaris mRNA in WHEEL + TREADMILL compared to WHEEL (Fig.3F), but not in the soleus (Fig.3E). We also compared the mRNA levels of GLUT4 and CPT1B in soleus and plantaris muscles, but no significant differences were detected (Fig.3A, B, G, and H).

**Figure 4:**
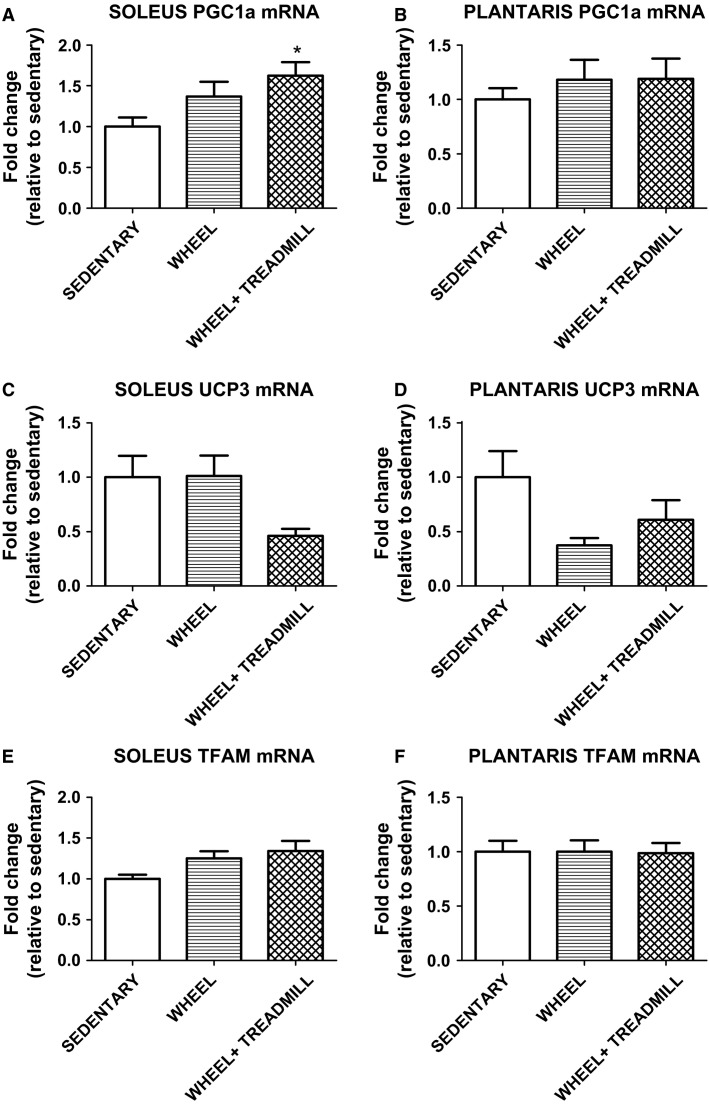
Expression of mRNA of select mitochondrial markers in soleus and plantaris. PGC-1α = peroxisome proliferator-activated receptor gamma coactivator 1-alpha; UCP3 = uncoupling protein 3; TFAM = mitochondrial transcription factor A. *Different from SEDENTARY. *n* = 6–7/group.

Furthermore, select mRNA markers of mitochondrial biogenesis were compared in the soleus and plantaris muscles. PGC-1a is involved in modulating many aspects of mitochondrial function including mitochondrial biogenesis. Soleus mRNA expression of PGC-1a was significantly increased in WHEEL + TREADMILL compared to SEDENTARY (*P* < 0.05), but there were no significant differences in PGC-1a mRNA expression in plantaris (Fig.4A and B). Another mechanism that mitochondrial biogenesis is enabled is by TFAM (Virbasius and Scarpulla 1994). Specifically, TFAM is a nuclear-encoded protein that is required to be imported into the mitochondria to promote mitochondrial DNA (mtDNA) transcription (Gordon et al. 2001). However, our data reveal that there were no significant differences in TFAM after training in soleus and plantaris muscles (Fig.4E and F). Another important marker of mitochondrial function is UCP3. Specifically, UCP3 is expressed mainly in skeletal muscle and its function is generally associated with energy metabolism (Schrauwen and Hesselink 2002). No significant differences were detected in plantaris and soleus mRNA levels of UCP3 (Fig.4C and D).

### Antioxidant enzymes and oxidative damage

**Figure 5:**
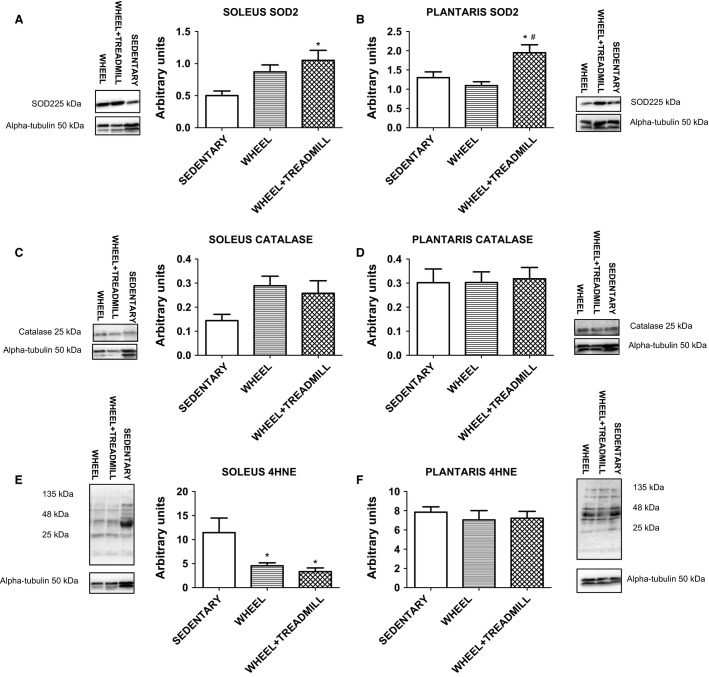
Antioxidant protein levels and marker of oxidative damage in soleus and plantaris. Representative western blot images are shown to the left-hand (for soleus) and to the right-hand (for plantaris) sides of the graphs. SOD2 = superoxide dismutase 2; 4HNE = 4-hydroxynonenal. *Different from SEDENTARY. ^#^Different from WHEEL. *n* = 6–7/group.

Enzymatic antioxidant proteins combat against oxidative damage (Powers et al. 2010, 2011). SOD2 protein levels were significantly (*P* < 0.05) higher in WHEEL + TREADMILL solei compared to SEDENTARY solei (Fig.5A). Also, in plantaris SOD2 protein levels were significantly (*P* < 0.05) higher in WHEEL + TREADMILL animals compared to SEDENTARY and WHEEL groups (Fig.5B). No significant differences existed for catalase protein levels in soleus or plantaris (Fig.5C and D). Furthermore, we measured the levels of oxidative damage in soleus and plantaris from the three groups by 4HNE. Interestingly, groups with access to a running wheel showed markedly decreased (*P* < 0.05) levels of 4HNE in the soleus (Fig.5E), but no significant differences existed in plantaris (Fig.5F).

## Discussion

In this study, we used a unique polygenic model of rat physical activity and the key finding is that while low levels of physical activity reduce body mass in low voluntary wheel running rats, additional exercise interventions (i.e., rats performing an additional treadmill exercise regimen 5 days/week) can elicit beneficial skeletal muscle adaptations, as well as potentiate voluntary physical activity. The 5-week duration of this study extends on current knowledge of low voluntary wheel running rodents after 6 days of wheel running (Roberts et al. 2013) and is the first study to demonstrate adaptive responses to extended duration exercise participation in this important animal model. Specific details of these findings are discussed in the following paragraphs.

While it is challenging to relate physiological changes between rodents and humans, the low voluntary wheel running paradigm is gaining attention as a model that provides mechanistic insight into the fact that inherently active and inactive behaviors are polygenic in nature. Importantly, the creation of the low voluntary wheel running model is an important facet of understanding the ongoing trend and resulting consequences of sedentary lifestyles occurring throughout the U.S. Our data demonstrate that even moderate levels of physical activity are sufficient to decrease body mass compared to sedentary behavior. Both groups with access to a wheel had significantly lower body mass in comparison to SEDENTARY, and this finding does not depend on a remodeling of the muscle. Although body mass changes occurred in animals with access to a wheel, no myosin heavy chain isotype differences existed between the SEDENTARY and WHEEL animals in soleus and plantaris muscles. When animals with access to a wheel were also exercised on the treadmill (WHEEL + TREADMILL), a myosin heavy chain shift was observed in the plantaris from a glycolytic fiber type to a more oxidative fiber type. Interestingly, the added stimulus of treadmill training resulted in increased participation in voluntary wheel running for WHEEL + TREADMILL rats. We speculate that the increased voluntary physical activity in WHEEL + TREADMILL could be indicative of several factors, including (1) decrease in relative difficulty performing exercise due to skeletal muscle adaptations from training, (2) alterations in the intrinsic mechanisms that drive exercise motivation, (3) increased sensitivity of reward–response relationship, and (4) stress and/or hormonal changes due to treadmill running. While this study does not approach matters of the latter two points, previous studies have observed the physiological differences that alter motivation in participation in voluntary running in these animals.

We posited that the increasing levels of physical activity via addition of treadmill training would result in increased markers of metabolic capacities. In a previous study, we observed no differences in inherent mRNA levels between animals bred to run high voluntary distances and animals bred to run low voluntary distances for plantaris and soleus muscles in PGC-1α, GLUT4, and VEGF after 6 days of voluntary wheel running (Roberts et al. 2013). Thus, a directive of this study was to broaden the scope of metabolic expression patterns elicited by an extended exercise intervention and to observe what, if any, benefits are mediated by addition of treadmill training to low voluntary wheel running rats. No differences were detected in soleus and plantaris GLUT4 mRNA. CPT1B mRNA also demonstrated no differences between groups in this current study. However, gene expression changes were present in FNDC5, a key signaling myokines that induce metabolic effects. Observation of skeletal muscle gene expression shows that FNDC5 mRNA was increased in plantaris of WHEEL + TREADMILL animals. FNDC5 is an upstream mediator of irisin and can result in the browning of adipose tissue (Bostrom et al. 2012). Brown adipose tissue increases energy expenditure in the form of heat and is important in thermogenesis. Irisin is proposed to be PGC-1α-dependent and thus is affected by exercise. Increased inefficiency in the form of increased heat production could actually be beneficial for overweight individuals who need to increase energy expenditure to aid in weight loss. However, the role of FNDC5 on metabolism in exercise scenarios needs to be further investigated. For example, Bostrom et al. (2012) observed that exercise training elicited increased FNDC5 levels in humans and rats following exercise, and Xu et al. (2011) demonstrated browning of adipose tissue after exercise. However, other investigators did not observe changes in brown adipose tissues in humans exercising for 12 weeks (Norheim et al. 2014). Thus, future research should continue to examine the broader physiological role and/or functional outcomes of exercise-induced FNDC5 expression, and in particular with respect to animal models where genetic predispositions appear to be closely linked to phenotype.

Endurance exercise promotes mitochondria biogenesis and results in a subsequent increase in muscle oxidative capacity. On the other hand, lifetime sedentary behavior in mice causes decreased mitochondrial function and a transient increase in oxidative damage (Figueiredo et al. 2009). Considering that mitochondria dysfunction is linked with several metabolic and cardiovascular diseases (Petersen et al. 2003), it is important to understand which type of physical activity impacts key mitochondrial genes. For example, PGC-1α is one of the most important regulators of mitochondrial biogenesis by inducing mtDNA replication and transcription via downstream proteins such as uncoupling proteins and nuclear respiratory factors (Wu et al. 1999; Vega et al. 2000). In the current study, PGC-1α mRNA expression was significantly higher in the soleus of WHEEL + TREADMILL in comparison to SEDENTARY. This is an interesting finding when compared to the changes that occurred in the plantaris FNDC5 mRNA described above and warrants further investigation why we observed these differences in these two different muscles. Nevertheless, increasing PGC-1a expression has been demonstrated to create a driving force of fiber-type shift toward more oxidative fiber types (Lin et al. 2002). While there was no fiber-type differences detected in soleus muscles, plantaris muscles of WHEEL + TREADMILL exhibited decreased Type IIx/b fibers indicating a fiber-type shift to the more oxidative Type IIa fiber type. In conjunction with this evidence, we speculate that adaptations most likely occurred at an earlier time course in plantaris of WHEEL + TREADMILL, a more glycolytic muscle fiber type. Conversely, elevated mRNA expression of PGC-1a in WHEEL + TREADMILL may indicate that soleus muscle could undergo a fiber-type shift as well, but requires a larger volume of training due to the inherent oxidative fiber type in soleus.

While the production of reactive oxygen species is a naturally occurring phenomenon and required for skeletal muscle adaptations, imbalance between oxidants and the defense system of antioxidants can lead to oxidative damage (Powers et al. 2010, 2011). As exercise is known to elicit increased release of free radicals, a corresponding increase in endogenous antioxidant proteins accompanies chronic exercise (Powers et al. 1999). In the current study, we measured the protein levels of two primary antioxidant enzymes (SOD2 and catalase) and a marker of oxidative damage (4HNE). The SOD2 enzyme acts to convert superoxide anions into oxygen and hydrogen peroxide, which is further broken down into water and oxygen by catalase. WHEEL + TREADMILL animals had higher levels of SOD2 in soleus and plantaris. Importantly, all groups of animals with access to a running wheel had significantly lower levels of soleus 4HNE indicating a decrease in oxidative damage. These data suggest that although additional stimuli for a low physically active population may be needed to obtain significant antioxidant protein adaptations in skeletal muscle, even low daily physical activity can mitigate the amount of oxidative damage apparent in a sedentary lifestyle. While beyond the scope of the current study, our interpretation of redox protection in all running wheel groups can be further extrapolated into the potential role that oxidative damage plays in atrophy, disease, and aging. In cases of muscle disuse, such as a sedentary individual, oxidants are known to activate proteolytic pathways causing protein degradation that leads to muscle atrophy (Powers et al. 2005, 2007). Additionally, an increase in oxidants is associated with aging and mitochondrial dysfunction (Lin and Beal 2006).

In conclusion, human trials to determine the effects that varying levels of physical activity have on the body are multifaceted, expensive, and difficult to perform. In a society where the percentage of people meeting the recommend physical activity guidelines is the minority, observing the optimal “minimal” amount of formal exercise at which beneficial physiological adaptations occur could serve to set a standard, in which meeting that threshold could improve the lives of millions. While extrapolating findings of physiological value from rodents to humans remains challenging, we propose the current rat model naturally mimics aspects of low voluntary participation in exercise in humans. The results from the experiments performed currently demonstrate that weight gain was attenuated in animals given access to a running wheel for 5 weeks. Interestingly, the addition of treadmill training increased the volume of voluntary physical activity, suggesting alterations in perceived difficulty or intrinsic motivational factors (Garland et al. 2011; Roberts et al. 2014). Furthermore, our data from strategic biomarkers for mitochondria, antioxidants, and metabolism indicate the usefulness of adding formal exercise in order to induce adaptations in skeletal muscle. These findings lay the ground work for future study on the rodent model and continue to support the expanding knowledge base that physical activity is necessary for a healthy phenotype.

## Conflict of Interest

None declared.
